# Extracellular matrix-derived scaffolds in constructing artificial ovaries for ovarian failure: a systematic methodological review

**DOI:** 10.1093/hropen/hoad014

**Published:** 2023-04-20

**Authors:** Tong Wu, Ke-Cheng Huang, Jin-Feng Yan, Jin-Jin Zhang, Shi-Xuan Wang

**Affiliations:** National Clinical Research Center for Obstetrical and Gynecological Diseases, Tongji Hospital, Tongji Medical College, Huazhong University of Science and Technology, Wuhan, China; Key Laboratory of Cancer Invasion and Metastasis, Ministry of Education, Tongji Hospital, Tongji Medical College, Huazhong University of Science and Technology, Wuhan, China; Department of Obstetrics and Gynecology, Tongji Hospital, Tongji Medical College, Huazhong University of Science and Technology, Wuhan, China; National Clinical Research Center for Obstetrical and Gynecological Diseases, Tongji Hospital, Tongji Medical College, Huazhong University of Science and Technology, Wuhan, China; Key Laboratory of Cancer Invasion and Metastasis, Ministry of Education, Tongji Hospital, Tongji Medical College, Huazhong University of Science and Technology, Wuhan, China; Department of Obstetrics and Gynecology, Tongji Hospital, Tongji Medical College, Huazhong University of Science and Technology, Wuhan, China; National Clinical Research Center for Obstetrical and Gynecological Diseases, Tongji Hospital, Tongji Medical College, Huazhong University of Science and Technology, Wuhan, China; Key Laboratory of Cancer Invasion and Metastasis, Ministry of Education, Tongji Hospital, Tongji Medical College, Huazhong University of Science and Technology, Wuhan, China; Department of Obstetrics and Gynecology, Tongji Hospital, Tongji Medical College, Huazhong University of Science and Technology, Wuhan, China; School of Materials Science and Engineering, Huazhong University of Science and Technology, Wuhan, China; National Clinical Research Center for Obstetrical and Gynecological Diseases, Tongji Hospital, Tongji Medical College, Huazhong University of Science and Technology, Wuhan, China; Key Laboratory of Cancer Invasion and Metastasis, Ministry of Education, Tongji Hospital, Tongji Medical College, Huazhong University of Science and Technology, Wuhan, China; Department of Obstetrics and Gynecology, Tongji Hospital, Tongji Medical College, Huazhong University of Science and Technology, Wuhan, China; National Clinical Research Center for Obstetrical and Gynecological Diseases, Tongji Hospital, Tongji Medical College, Huazhong University of Science and Technology, Wuhan, China; Key Laboratory of Cancer Invasion and Metastasis, Ministry of Education, Tongji Hospital, Tongji Medical College, Huazhong University of Science and Technology, Wuhan, China; Department of Obstetrics and Gynecology, Tongji Hospital, Tongji Medical College, Huazhong University of Science and Technology, Wuhan, China

**Keywords:** decellularization, artificial ovary, ovarian failure, extracellular matrix, ovarian tissue transplantation, methodology

## Abstract

**STUDY QUESTION:**

What is the current state-of-the-art methodology assessing decellularized extracellular matrix (dECM)-based artificial ovaries for treating ovarian failure?

**SUMMARY ANSWER:**

Preclinical studies have demonstrated that decellularized scaffolds support the growth of ovarian somatic cells and follicles both *in vitro* and *in vivo*.

**WHAT IS KNOWN ALREADY:**

Artificial ovaries are a promising approach for rescuing ovarian function. Decellularization has been applied in bioengineering female reproductive tract tissues. However, decellularization targeting the ovary lacks a comprehensive and in-depth understanding.

**STUDY DESIGN, SIZE, DURATION:**

PubMed, Embase, Web of Science, and the Cochrane Central Register of Controlled Trials were searched from inception until 20 October 2022 to systematically review all studies in which artificial ovaries were constructed using decellularized extracellular matrix scaffolds. The review was performed according to the Preferred Reporting Items for Systematic Reviews and Meta-Analyses (PRISMA) protocol.

**PARTICIPANTS/MATERIALS, SETTING, METHODS:**

Two authors selected studies independently based on the eligibility criteria. Studies were included if decellularized scaffolds, regardless of their species origin, were seeded with ovarian cells or follicles. Review articles and meeting papers were removed from the search results, as were articles without decellularized scaffolds or recellularization or decellularization protocols, or control groups or ovarian cells.

**MAIN RESULTS AND THE ROLE OF CHANCE:**

The search returned a total of 754 publications, and 12 papers were eligible for final analysis. The papers were published between 2015 and 2022 and were most frequently reported as coming from Iran. Detailed information on the decellularization procedure, evaluation method, and preclinical study design was extracted. In particular, we concentrated on the type and duration of detergent reagent, DNA and extracellular matrix detection methods, and the main findings on ovarian function. Decellularized tissues derived from humans and experimental animals were reported. Scaffolds loaded with ovarian cells have produced estrogen and progesterone, though with high variability, and have supported the growth of various follicles. Serious complications have not been reported.

**LIMITATIONS, REASONS FOR CAUTION:**

A meta-analysis could not be performed. Therefore, only data pooling was conducted. Additionally, the quality of some studies was limited mainly due to incomplete description of methods, which impeded specific data extraction and quality analysis. Several studies that used dECM scaffolds were performed or authored by the same research group with a few modifications, which might have biased our evaluation.

**WIDER IMPLICATIONS OF THE FINDINGS:**

Overall, the decellularization-based artificial ovary is a promising but experimental choice for substituting insufficient ovaries. A generic and comparable standard should be established for the decellularization protocols, quality implementation, and cytotoxicity controls. Currently, decellularized materials are far from being clinically applicable to artificial ovaries.

**STUDY FUNDING/COMPETING INTEREST(S):**

This study was funded by the National Natural Science Foundation of China (Nos. 82001498 and 81701438). The authors have no conflicts of interest to declare.

**TRIAL REGISTRATION NUMBER:**

This systematic review is registered with the International Prospective Register of Systematic Reviews (PROSPERO, ID CRD42022338449).

WHAT DOES THIS MEAN FOR PATIENTS? Ovarian failure will not only lead to fertility loss but can also affect a woman’s overall health and quality of life. One of the strategies under development to treat ovarian failure is the construction of artificial ovaries by encapsulating healthy ovarian cells or follicles into ovarian scaffolds. Decellularized scaffolds are created by the removal of the cells while preserving the natural tissue matrix; they have been widely studied in tissue regeneration research and have been applied in clinical practice. This systematic review was conducted to evaluate whether the decellularization-based artificial ovary can be used to restore ovarian function. Specifically, we provide detailed information on the ovarian decellularization procedure, evaluation method, and preclinical study design. The decellularized scaffolds loaded with ovarian cells can produce estrogen and progesterone, though with high variability, and support the growth of follicles, without reports of serious complications. Thus, the decellularization-based artificial ovary may be a promising choice for restoring ovarian function and improving female fertility in the future.

## Introduction

Ovarian failure is characterized by the disruption of both endocrine and reproductive ovarian function and has gained increased interest in reproductive medicine, oncofertility, and organ aging (Mauri *et al.*, 2020). Ovarian failure can be attributed to chemotherapy, radiotherapy, natural aging, or genetic predisposition (Sükür *et al.*, 2014; Qin *et al.*, 2015; Chemaitilly *et al.*, 2017; Cui *et al.*, 2018). An exhausted ovary will not only lead to fertility loss but can also increase the risks of cardiovascular disease, osteoporosis, and urogenital atrophy (Proserpio *et al.*, 2020). As the average lifespan of women has exceeded 80 years, a naturally menopausal woman will spend almost 40% of her lifetime in the post-menopausal phase (Takahashi and Johnson, 2015). However, young women with primary ovarian insufficiency experience menopause even earlier. Ovarian failure can considerably affect a woman’s overall health, work productivity and quality of life. Accordingly, minimizing the adverse effects of ovarian failure is important and urgent.

Common treatments for ovarian failure include pharmacological medication, which mainly involves the supplementation of estrogen alone or estrogen–progestogen combinations. However, hormone replacement therapy should be implemented with particular caution, as the dosage, frequency, and time frame require individualization (Kling *et al.*, 2019; Flores *et al.*, 2021). Novel strategies such as ovarian tissue cryopreservation (OTC) and ovarian tissue transplantation (OTT) have arisen over the last two decades (Donnez *et al.*, 2004), particularly for restoration of fertility after cancer treatments. To date, OTC and OTT have led to 189 deliveries and have been shown to restore ovarian function for many years (Donnez and Dolmans, 2017, 2018; Khattak *et al.*, 2022). However, some groups and countries, such as the American Society of Clinical Oncology, consider OTC an experimental technique (Oktay *et al.*, 2018). The method is also hampered by the risk of tumor reoccurrence due to hidden malignant cells within tissue grafts (Stern *et al.*, 2014; Fajau-Prevot *et al.*, 2017).

An artificial ovary is also a promising approach for rescuing ovarian function (Cho *et al.*, 2019). It is constructed by encapsulating healthy ovarian cells or follicles in scaffolds to replace failed ovaries (Amorim and Shikanov, 2016). Various polymers have been utilized to create scaffolds to fabricate biomimetic functional ovaries. Synthetic polymers, such as gelatin-methacryloyl and polyethylene glycol, are easily manufactured and show enhanced mechanical properties but have limited cell adhesion sites (Mendez *et al.*, 2018; Wu *et al.*, 2022a). In contrast, alginate and fibrin are the two most commonly used natural materials as they have a large diversity of integrin-binding motifs and are more biocompatible. Decellularized ovaries are also based on these individual extracellular matrix (ECM) components and have the advantage of retaining the tissue inner spatial distribution of the tissue as well as its vascularization channels and mechanical properties. Decellularized ovaries are therefore attracting much attention in the field of organ regeneration (Kim *et al.*, 2021b).

**Figure 1.**
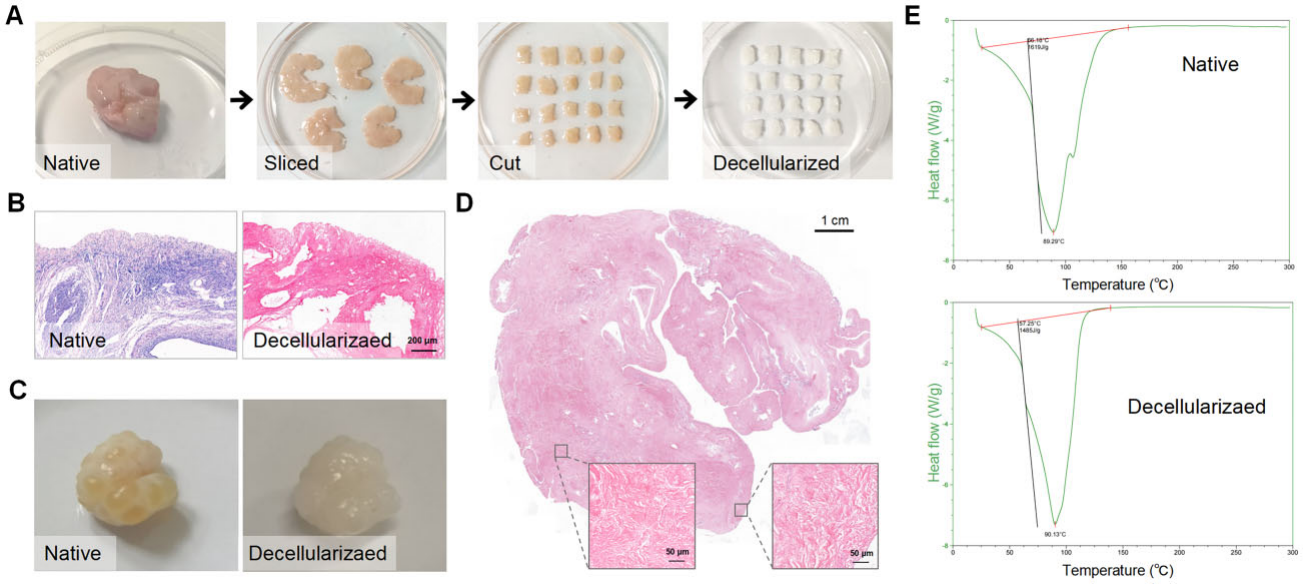
**The process and evaluation of extracellular matrix (ECM)-based scaffolds.** (**A**) The color of the ovarian cortical strips turns from red to white after decellularization but displays comparable shapes. (**B**) H&E staining of the ovarian cortical strips. (**C**) The change in an intact porcine ovary after decellularization. (**D**) H&E staining shows the absence of basophilic materials in the decellularized tissues. (A–D) Reprinted with permission from Wu *et al.* (2022b). (**E**) Representative images of thermal analysis characterization; the different slopes indicate the change of ECM ultrastructure or motifs after decellularization. ECM, extracellular matrix; H&E, hematoxylin and eosin.

Decellularization refers to the removal of the cellular compartments while preserving the natural ECM with optimal porosity, stiffness and elasticity, thus yielding decellularized ECM (dECM) constructs (Fig. 1) (Saldin *et al.*, 2017; Wu *et al.*, 2022b). Decellularization has been widely studied in bone, heart, dermal tissues, and small intestinal submucosa, in both basic research and clinical practice (Bejleri and Davis, 2019; Xu *et al.*, 2020; Datta *et al.*, 2021). For example, commercial dECM products have been approved for pericardial reconstruction and have demonstrated good performance (van Rijswijk *et al.*, 2020; Mohamed *et al.*, 2021). Acellular dermal matrices are also available to promote breast reconstruction following mastectomy for breast cancer (Folli *et al.*, 2018). Similar to the aforementioned dECM materials, decellularized ovarian scaffolds can also provide tissue-specific biomechanical cues to facilitate cell growth and can therefore be an ideal platform to support follicular development and restoration of ovarian function (Hoshiba, 2021). This encouraging method has been commonly discussed in female reproduction bioengineering topics (Gandolfi *et al.*, 2020; Kim *et al.*, 2021b; Francés-Herrero *et al.*, 2022). However, a comprehensive and in-depth understanding of bioengineered ovaries is currently lacking. Since the first successful decellularization of human and bovine ovarian tissues in 2012, an increasing number of relevant papers has been published in recent years (Laronda *et al.*, 2015). As many more different strategies have been proposed, there is an urgent need to compile previous work and to guide future studies.

This systematic review summarizes the recent progress in constructing artificial ovaries based on dECM scaffolds, elucidates its application in restoring ovarian function, and provides a theoretical basis for future optimizations and improvements.

## Materials and methods

### Protocol and registration

This systematic review was registered with the International Prospective Register of Systematic Reviews (PROSPERO, ID: CRD42022338449) and was performed according to the Preferred Reporting Items for Systematic Reviews and Meta-Analyses (PRISMA) protocol (Moher *et al.*, 2009). This systematic review aims to answer the question ‘What is the current state-of-the-art methodology to assess dECM-based artificial ovaries for treating ovarian failure?’ The search terms were based on a PICO (population, intervention, comparison and outcome) framework: animals and humans (P) with dECM-based artificial ovaries (I) as compared with controls (C) to support follicular growth or restore ovarian function (O) (Schardt *et al.*, 2007).

### Literature search

A systematic search was conducted in the PubMed, Embase, Web of Science, and the Cochrane Central Register of Controlled Trials electronic medical databases from inception until 20 October 2022. We used the following search keywords to maximally cover the relevant literature: ‘decellularization’, ‘acellular’, ‘recellularization’, ‘ovary’, ‘ovarian tissue’, and ‘follicle’ (Supplementary Table S1). Articles were identified using MeSH headings and keywords combined with Boolean operators. There was no restriction on the date or publication status.

### Eligibility criteria

Studies were included if the decellularized scaffolds were seeded with ovarian cells or follicles. For example, scaffolds derived from amniotic membranes and loaded with ovarian cells were included. Studies were excluded if: (i) the ovarian dECM scaffolds were not loaded with ovarian cells, (ii) the decellularization protocol was not described, (iii) the scaffold was not obtained by decellularization, (iv) no control group was established, (v) it was a repeated record, or (vi) it was a non-English language paper. Studies describing the changes before and after decellularization without control groups were excluded because the alterations might be confounded by placebo effects, or research bias or other changes in the experiment site (Grimes and Schulz, 2002). Different original articles by the same group using the same decellularization method were defined as repeated records and only the most recent version was retained.

### Study selection and data collection

**Figure 2.**
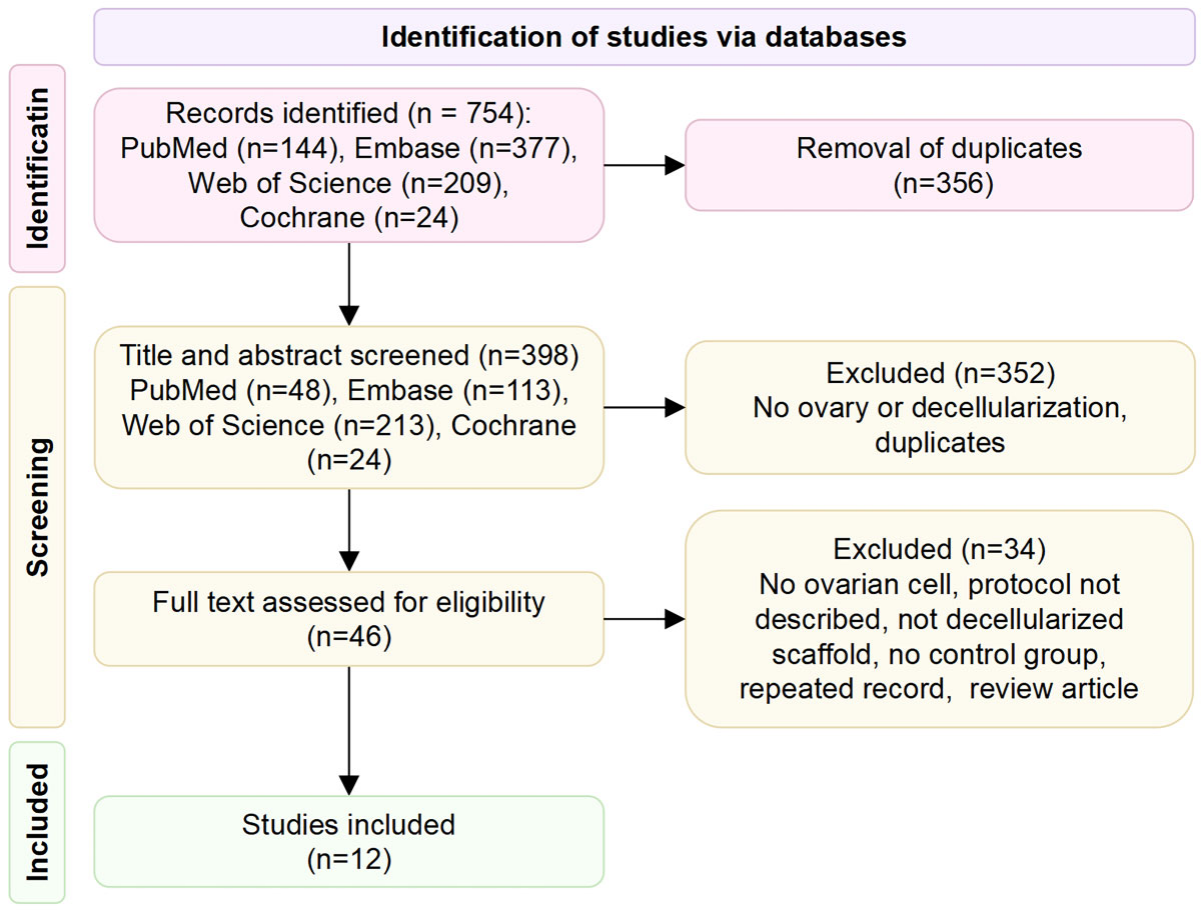
Flow diagram of study selection on 20 October 2022.

Two reviewers (T.W. and K.-C.H.) independently searched the electronic medical databases and selected studies based on the eligibility criteria (Fig. 2). Discrepancies between the selected studies by both authors were discussed in a consensus meeting with the senior authors (J.-J.Z. and S.-X.W.) providing a binding verdict.

### Data extraction

Data were independently extracted by two reviewers (T.W. and J.-F.Y.). The outcomes of interest covered most ovary decellularization details and were classified into three groups: decellularization procedure, evaluation method, and preclinical study design. Specifically, the following information was recorded: author, publication year, country, animal species, preprocessing program, type and duration of detergent reagent and enzyme, biocompatibility, DNA and ECM detection methods, seeding cells, experimental grouping, and main findings.

The risk of bias was assessed by two independent blinded reviewers (T.W. and J.-F.Y.) using the SYstematic Review Centre for Laboratory animal Experimentation (SYRCLE) tool (Hooijmans *et al.*, 2014). The SYRCLE tool addresses selection bias, performance bias, attrition bias, detection bias and reporting bias. Each item of a study is assigned ‘yes’ (low risk of bias), ‘no’ (high risk of bias), or ‘unclear’ (insufficient details).

## Results

### Included articles

The initial electronic database search returned 754 papers, of which 398 remained after duplicates had been removed. There were 46 full-text studies remaining for assessment of the eligibility criteria. After full-text screening, 34 studies were excluded as they did not meet the inclusion criteria and 12 studies were included (Fig. 2, Supplementary Table S2). All 12 studies performed decellularization and recellularized dECM scaffolds with cells and compared them with the control groups. The studies were appraised for risk of bias (Supplementary Table S3). The allocation concealment was unclear in all studies, and the lack of information on housing of animals and observer blinding to the interventions resulted in a risk of performance bias. The attrition bias was either low risk (n = 5) or unclear (n = 7) in the included studies. The risks of selective reporting and other biases were low for most studies. The studies were published between 2015 and 2022 and were conducted in five countries, most frequently in Iran (n = 7), followed by China (n = 2), Belgium (n = 1), the USA (n = 1), and Italy (n = 1).

### Characteristics of decellularization approaches

Of the 12 papers, 10 used one species (3 studies each used human and porcine tissue, respectively, 2 studies used mouse tissue, and 1 study each used bovine and sheep tissue, respectively). Two studies utilized tissues from humans and other species simultaneously (Table 1). The most common source of dECM scaffolds was ovarian tissues (n = 8), followed by amniotic membranes (n = 2), greater omentum (n = 1), and peritoneal membranes (n = 1) (Motamed *et al.*, 2017; Sarabadani *et al.*, 2021; Haghshenas *et al.*, 2022). In seven studies, different regions of tissues (six ovaries and one amniotic membrane) were dissected and subsequently minced (Supplementary Table S4) (Laronda *et al.*, 2015; Motamed *et al.*, 2017; Hassanpour *et al.*, 2018; Nikniaz *et al.*, 2021; Chiti *et al.*, 2022; Zheng *et al.*, 2022; Wu *et al.*, 2022b). The greater omentum and amniotic membrane were directly cut into small fragments (Fazelian-Dehkordi *et al.*, 2022; Haghshenas *et al.*, 2022). Four studies conducted freeze–thaw cycles. Fazelian-Dehkordi *et al.*, (2022) performed the most complicated preprocessing, with six steps that spanned more than 2 days.

**Table 1.** Characteristics of decellularization procedures.

Study ID	Species	Tissue	Detergent reagent	Enzyme
Alaee *et al.* (2021)	Mouse	Ovary	1% SLES (4 h)	Not used
Chiti *et al.* (2022)	Bovine	Ovary	3% Triton X-100 (1 h) 4% SDC (1 h)	0.05% trypsin/0.02% EDTA (1 h)
Fazelian-Dehkordi *et al.* (2022)	Sheep	Great omentum	1% SDS (48 h) 2.5 mM SDC (24 h)	Not used
Haghshenas *et al.* (2022)	Human	Amniotic membrane	0.1% SDS (24 h) ddH2O (48 h)	Not used
Hassanpour *et al.* (2018)	Human	Ovary	1% SLES for cortex (48 h) 1% SLES for intact ovary (30–40 days)	500 U/ml DNase I (24 h)
Laronda *et al.* (2015)	Human and bovine	Ovary	0.1% SDS for cortex (24 h) 0.1% SDS for intact ovary (21 days)	Not used
Motamed *et al.* (2017)	Human	Amniotic membrane	Not used	0.25% trypsin/0.02% EDTA (2 h)
Nikniaz *et al.* (2021)	Human and porcine	Ovary	0.5% SDS (2 h) 1% Triton X-100/0.1% ammonium hydroxide (22 h)	Not used
Pennarossa *et al.* (2021)	Porcine	Ovary	0.5% SDS (3 h) 1% Triton X-100 (18 h) 2% SDC (18 h)	Not used
Sarabadani *et al.* (2021)	Mouse	Peritoneal membrane	5% trypsin/EDTA (1.5 h) 2-propanol (10 h)	Trypsin/EDTA (2 h) 0.25% trypsin/0.1% EDTA (4 h)
Wu *et al.* (2022b)	Porcine	Ovary	2% SDC/4% Triton X-100 (36 h) 1% Triton X-100 (36 h)	RNase/DNase 80 U/ml (6 h)
Zheng *et al.* (2022)	Porcine	Ovary	0.1% SDS/PMSF (12 h) 1% Triton X-100 (7 days)	50 U/ml DNase I/1 U/ml RNase (12.5 h)

EDTA, ethylene diamine tetraacetic acid; SDC, sodium deoxycholate; SDS, sodium dodecyl sulfate; SLES, sodium lauryl ester sulfate; PBS, phosphate-buffered saline; PMSF, phenylmethylsulfonyl fluoride.

Decellularization is greatly affected by detergent type, exposure time, and incubation temperature. Balancing these factors to sufficiently remove cells and preserve the ECM is vital for decellularization. Four studies used solo chemicals for decellularization, where two studies each used sodium dodecyl sulfate (SDS) and sodium lauryl ester sulfate (SLES), respectively. Seven studies used detergent combinations (Nikniaz *et al.*, 2021; Pennarossa *et al.*, 2021; Sarabadani *et al.*, 2021; Chiti *et al.*, 2022; Fazelian-Dehkordi *et al.*, 2022; Zheng *et al.*, 2022; Wu *et al.*, 2022b). From the perspective of chemical types, ionic detergents (SDS, sodium deoxycholate, SLES) were the most commonly used reagents (n = 10) (Laronda *et al.*, 2015; Hassanpour *et al.*, 2018; Alaee *et al.*, 2021; Nikniaz *et al.*, 2021; Pennarossa *et al.*, 2021; Chiti *et al.*, 2022; Haghshenas *et al.*, 2022; Zheng *et al.*, 2022; Wu *et al.*, 2022b), and five studies used non-ionic detergents, mainly Triton X-100 (Nikniaz *et al.*, 2021; Pennarossa *et al.*, 2021; Chiti *et al.*, 2022; Zheng *et al.*, 2022; Wu *et al.*, 2022b). Less frequently used detergents included ammonium hydroxide and 2-propanol. Trypsin/EDTA and DNase/RNase solution were equally frequently used enzymes following detergents (n = 6) (Motamed *et al.*, 2017; Hassanpour *et al.*, 2018; Sarabadani *et al.*, 2021; Chiti *et al.*, 2022; Zheng *et al.*, 2022; Wu *et al.*, 2022b). In three studies, the dECM scaffolds were further processed into hydrogels, which were easier to use and apply (Supplementary Table S4) (Chiti *et al.*, 2022; Haghshenas *et al.*, 2022; Zheng *et al.*, 2022). Regarding the operation duration, most studies completed decellularization within several days, except for those that used intact ovaries, where the operation spanned >3 weeks (Laronda *et al.*, 2015; Hassanpour *et al.*, 2018).

### Efficiency evaluation methods

The accepted standard for evaluating decellularization efficacy is: (i) maximal 50 ng/mg DNA per dry weight ECM, (ii) maximum 200-bp DNA fragment size, and (iii) negative histology for nuclear materials in tissues stained with 4′,6-diamidino-2-phenylindole (DAPI) or hematoxylin and eosin (H&E) (Crapo *et al.*, 2011). The DNA content within scaffolds was qualitatively described and quantitatively detected in all 12 studies (Table 2). However, DNA content >50 ng/mg was observed in two studies, which indicated insufficient decellularization (Pennarossa *et al.*, 2021; Haghshenas *et al.*, 2022). Resident ECM proteins were observed using connective tissue staining, such as Masson trichrome, Alcian blue, and periodic acid-Schiff staining, in seven studies. Collagen type I and IV, laminin, and fibronectin were analyzed by immunological methods in three studies (Laronda *et al.*, 2015; Hassanpour *et al.*, 2018; Wu *et al.*, 2022b). Glycosaminoglycans were quantitatively determined in four studies (Motamed *et al.*, 2017; Chiti *et al.*, 2022; Fazelian-Dehkordi *et al.*, 2022; Haghshenas *et al.*, 2022). Other staining techniques such as Gomori (n = 2), Heidenhain’s Azan (n = 1), Mallory (n = 1) and orcein (n = 1) were also reported. In all studies, the ultrastructure was evaluated by scanning electron microscope (Supplementary Table S4). Raman spectrum was calculated in one study to analyze the DNA and ECM (Alaee *et al.*, 2021). Additionally, three studies tested the rheological properties of the dECM hydrogels (Chiti *et al.*, 2022; Haghshenas *et al.*, 2022; Zheng *et al.*, 2022). Eight studies routinely examined cytotoxicity and biocompatibility to rule out unwanted adverse effects (Motamed *et al.*, 2017; Hassanpour *et al.*, 2018; Nikniaz *et al.*, 2021; Pennarossa *et al.*, 2021; Fazelian-Dehkordi *et al.*, 2022; Haghshenas *et al.*, 2022; Zheng *et al.*, 2022; Wu *et al.*, 2022b).

**Table 2.** Evaluation of the decellularized extracellular matrix scaffolds.

Study ID	DNA examination		ECM examination	
	Description	Quantification (ng/mg)	Description	Quantification
Alaee *et al.* (2021)	H&E, Hoechst, Raman microscope	21.45 ± 3.36	MT, AB, Raman microscope	Not reported
Chiti *et al.* (2022)	Not reported	+ (dng)	Not reported	Collagen (not significant, 30.67 ± 0.2 μg/mg), GAG (dng)
Fazelian-Dehkordi *et al.* (2022)	H&E, Hoechst	<50	Aldehyde fuchsine, PAS, oil red, AB, methylene blue.	VEGF (500 ng/l), GAG (dng)
Haghshenas *et al.* (2022)	H&E, DAPI	114 ± 32.46	MT, AB	Collagen (not significant), GAG (decrease, 166.2 ± 5.87 μg/mg)
Hassanpour *et al.* (2018)	H&E, Hoechst	40 ± 7.33	Heidenhain’s AZAN, MT, Gomori, AB, IHC (COL1, COL4, LAM, FN)	Not reported
Laronda *et al.* (2015)	H&E, DAPI	+ (dng)	IF (COL1, COL4, LAM, FN)	Not reported
Motamed *et al.* (2017)	H&E	39.38 ± 4.04	MT	GAG (decrease, 43 ± 3.08 μg/mg)
Nikniaz *et al.* (2021)	H&E, DAPI	+ (dng)	Orcein, MT	Not reported
Pennarossa *et al.* (2021)	H&E, DAPI	50 ± 30	MT, Mallory, AB, Gomori	Not reported
Sarabadani *et al.* (2021)	H&E, DAPI	8.98	MT, PAS	Not reported
Wu *et al.* (2022b)	H&E, DAPI, gel electrophoresis	12.86 ± 1.707	MT, AB, PAS, sirius red, IHC (COL1, COL2, COL3, COL4, FN, LAM, AMH, TGFB, BMP15, CTGF)	Collagen (not significant), length, width, straightness of collagen
Zheng *et al.* (2022)	H&E, DAPI	48.48 ± 1.88	MT, Toluidine blue	Not reported

AB, Alcian blue; AMH: anti-Mullerian hormone; BMP, bone morphogenetic protein; COL, collagen; CTGF, connective tissue growth factor; DAPI, 4′,6-diamidino-2-phenylindole; dng, data not given; ECM, extracellular matrix; FN, fibronectin; H&E, hematoxylin and eosin; IF, immunofluorescence; IHC: immunohistochemistry; GAG, glycosaminoglycans; LAM, laminin; MT, Masson trichrome; PAS, periodic acid-Schiff; TGFB, transforming growth factor-β; VEGF, vascular endothelial growth factor.

### Functional restoration of dECM-based artificial ovaries

The ovarian scaffolds were successfully recellularized with follicles (n = 6), ovarian somatic cells (n = 5), cumulus–oocyte complex (n = 1), and epigenetically erased dermal fibroblasts (n = 1) (Supplementary Table S4). Mouse cells were most commonly used (n = 10). Eight groups cultured preantral follicles for 7–12 days on dECM scaffolds *in vitro* (Alaee *et al.*, 2021; Chiti *et al.*, 2022). The seeding cells or follicles were mainly assessed based on the follicle survival rate, growth diameter, antrum formation, oocyte maturation, hormone secretion, and mRNA/protein markers (Table 3) (Laronda *et al.*, 2015; Motamed *et al.*, 2017; Hassanpour *et al.*, 2018; Alaee *et al.*, 2021; Sarabadani *et al.*, 2021; Haghshenas *et al.*, 2022; Zheng *et al.*, 2022). Three studies demonstrated increased estradiol (E_2_) or progesterone (P_4_) (Motamed *et al.*, 2017; Alaee *et al.*, 2021; Sarabadani *et al.*, 2021). Four studies evaluated *in vivo* follicular development or ovarian function. Similar to the *in vitro* results, the dECM-based artificial ovaries contributed to higher E_2_ or P_4_ or inhibin A compared with those in ovariectomized mice (Laronda *et al.*, 2015; Hassanpour *et al.*, 2018; Zheng *et al.*, 2022). The molecular marker growth differentiation factor (GDF) 9 and GDF15, specifically expressed in oocytes, were the most studied markers (n = 2 each) (Motamed *et al.*, 2017; Alaee *et al.*, 2021; Pennarossa *et al.*, 2021). Altogether, all studies demonstrated the feasibility and benefits of dECM-based artificial ovaries.

**Table 3.** Preclinical study design of decellularized extracellular matrix scaffolds.

Study ID	Grouping	Main finding
Alaee *et al.* (2021)	① 2D culture ② dECM ③ Preantral follicle ④ *In vivo* matured	Higher rates of antral cavity formation and maturation in ② than ①. Higher levels of E_2_ and P_4_ in ② than ①. Survival rate and follicular diameter in ①②, nsd. Higher levels of *Zp2, Gdf9, Bmp6* and *Bmp15* in ③ than ①②④.
Chiti *et al.* (2022)	① Alginate ② 25% Alginate + 75% dECM ③ 10% Alginate + 90% dECM ④ dECM	Follicle recovery rate rose with increased alginate content. No follicles were recovered in ④. Follicle viability and growth in ①②③④, nsd.
Fazelian-Dehkordi *et al.* (2022)	① 2D dECM-FBS ② 2D dECM ③ 2D FBS ④ 2D control ⑤ 3D dECM-FBS ⑥ 3D dECM ⑦ 3D FBS ⑧ 3D control	More first polar body in the 3D groups containing dECM than other groups. More MII oocytes of 2D culture containing dECM than the 2D control groups. More nuclear maturation in 3D culture groups containing dECM than the 3D control group.
Haghshenas *et al.* (2022)	① Alginate ② Alginate + dECM 45 mg/mL ③ Alginate + dECM 30 mg/mL ④ Alginate + dECM 15 mg/mL	Higher antral follicles and lower follicle degeneration rate in ①② than ③④. Follicle diameter and E_2_ in ①②, nsd.
Hassanpour *et al.* (2018)	① Sham-operated ② OVX ③ OVX + dECM ④ OVX + dECM + OSC	The presence of immune cells and neovascularization in ③④. The distribution of INHa, ER and PR in ④, and DAZL in ③. Higher levels of E_2_ and P_4_ in ④ than ②③.
Laronda *et al.* (2015)	① Age-matched cycling mouse ② OVX + dECM ③ OVX + dECM + OSC	Higher levels of E_2_ and INHa in ①③ than ②. Comparable vaginal orifices time in ①③. Immune infiltration in ②③.
Motamed *et al.* (2017)	① Base medium ② Intact amniotic membrane ③ dECM	Higher follicular survival rate in ②③ than ①. Higher level of E_2_ in ② > ③ > ①. Higher ratio of *Bax/Bcl2* in ① than ②③. Higher levels of *Cx37*, *Gdf9* and *Bmp15* in ② than ①③.
Nikniaz *et al.* (2021)	① Alginate ② dECM	Higher ratio of follicular recovery in ② than ①.
Pennarossa *et al.* (2021)	① Native tissue ② dECM + OSC ③ dECM + porcine EpiE ④ dECM + human EpiE ⑤ Plastic + porcine EpiE ⑥ Plastic + human EpiE	Comparable levels of *Vim, Thy1, Star, Cyp11a1, Cyp19a1, Amh, Fshr* and *Lhr* in ①②. Comparable levels of *Star, Cyp11a1, Cyp19a1, Amh, Fshr* and *Lhr* in ②③④. Lower levels of *Vim* and *Thy1* in ③④ than ①.
Sarabadani *et al.* (2021)	① Base medium ② dECM ③ Base medium + peritoneal mesothelial stem cell ④ Conditioned medium	The viability of follicles in ①②③④, nsd. Larger follicle diameter in ③ than ①②④. More eccentric oocytes in ③ > ④ > ①②. Higher level of E_2_ in ③ > ①④ > ②.
Wu *et al.* (2022b)	① Sham-operated ② OVX ③ OVX + dECM ④ OVX + dECM + OSC/follicle	Follicles are able to growth *in vitro*. No estrus cycle restoration and vaginal orifice. The activation of immune cell infiltration and complement system.
Zheng *et al.* (2022)	① Sham-operated ② OVX ③ OVX + dECM ④ OVX + dECM + OSC ⑤ OVX + dECM hydrogel + OSC	More proliferating cells in ④ than ⑤. TUNEL-positive cells in ④⑤, nsd. Higher level of E_2_ in ④ than ②③. Restoration of FSH and P_4_ in ①④. More ER-, INHa- and FSH-positive cells in ④ than ⑤.

Bax, Bcl2 associated x; Bcl2, Bcl2 apoptosis regulator; Bmp, bone morphogenetic protein; Cyp, Cytochrome P450; DAPI, 4′,6-diamidino-2-phenylindole; DAZL, deleted in azoospermia like; dECM, decellularized extracellular matrix; E_2_, estradiol; EpiE, epigenetically erased dermal fibroblast; ER, estrogen receptor; FBS, fetal bovine serum; FSH, follicle stimulating hormone; Gdf, growth differentiation factor; GVBD, germinal vesicle breakdown; H&E, hematoxylin and eosin; INH, inhibin; nsd, not significant difference; MII, metaphase II; OSC, ovarian comatic cell; OVX, ovariectomy; P_4_, progesterone; PR, progestogen receptor; Star, steroidogenic acute regulatory; Thy1, Thy-1 cell surface antigen; TUNEL, terminal deoxynucleotidyl transferase-mediated dUTP nick end labeling; Vim, vimentin; ZP, zona pellucida glycoprotein.

## Discussion

**Figure 3.**
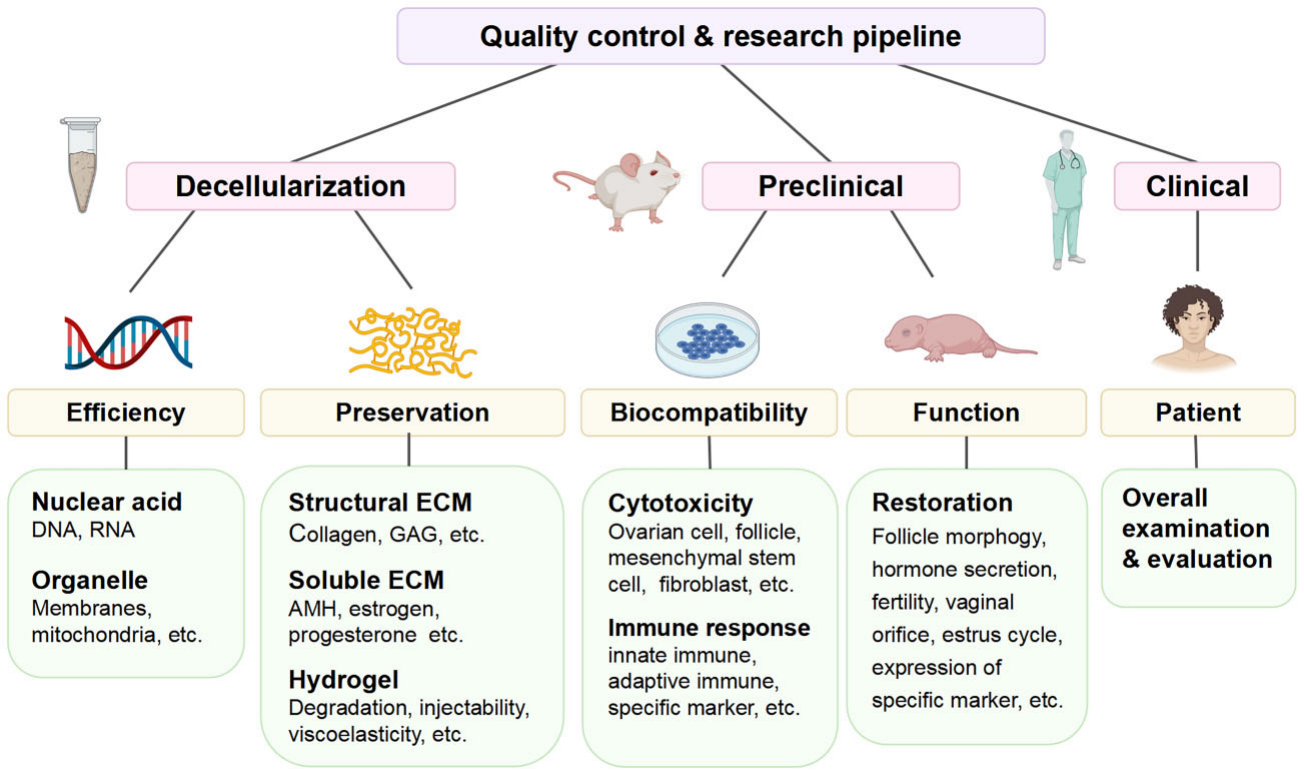
**Quality controls and clinical directions for decellularized ovaries.** AMH, anti-Mullerian hormone; DNA, deoxyribonucleic acid; ECM, extracellular matrix; GAG, glycosaminoglycan; RNA, ribonucleic acid.

This systematic review suggests that dECM materials hold promise for constructing artificial ovaries and counteracting ovarian failure. A total of 12 studies were included and the decellularization methods and evaluation parameters varied greatly. An optimal reproducible and standardized procedure is a prerequisite for future clinical application (Fig. 3) (Naso and Gandaglia, 2022). Both preclinical and clinical trials should comply with the quality control and research pipelines. For these reasons, the evaluations of the following characteristics of ovarian-specific dECM scaffolds are proposed: (i) DNA removal efficiency; (ii) ECM preservation; (iii) cell debris residues; (iv) biocompatibility; and (v) restoration of ovarian function.

Effective decellularization is reflected by the adequate removal of cellular components and good preservation of ECM proteins (Luo *et al.*, 2019). The tissue type, animal species, chemical reagents, and exposure time all affect the efficiency and should be balanced (Eivazkhani *et al.*, 2019). For example, minimizing the detergent concentration contributes to more retention of ECM, but it might also cause insufficient cell removal, risking a relevant immune response after *in vivo* implantation (Luo *et al.*, 2019; Chakraborty *et al.*, 2020). Regarding the DNA evaluation methods, it was interesting that only one study performed electrophoresis, while all studies conducted H&E/DAPI staining and DNA extraction. We speculated that this might be due to the complex manipulation processes compared with histological staining and DNA quantification (Lee *et al.*, 2012). However, neither DNA staining nor quantification can substitute for the evaluation of DNA size, as the two methods occasionally cannot detect the minimal virus DNA contents, which may nevertheless elicit an immune response (Chakraborty *et al.*, 2020). Furthermore, peracetic acid/ethanol treatment eliminates small DNA debris (Hodde and Hiles, 2002). Altogether, there should be more focus on comprehensive assessment of DNA residues, especially DNA fragments.

ECM is composed of structural and soluble components. The former includes collagen, laminin, fibronectin and elastin (Naba *et al.*, 2017; Yuzhalin *et al.*, 2018). The Masson trichrome and Heidenhain’s Azan staining method mainly detects fibrillar collagens (Polat *et al.*, 2007). Mallory staining can distinguish collagens from elastin (Chambrone *et al.*, 2015), while the Alcian blue staining is specific for glycosaminoglycans (Mead, 2020). Immunological methods can reveal the ECM protein distribution via antigen-antibody binding. However, the fact that various methods serve similar purposes and produce repeated results remains an issue. Most studies neglect other equally important molecules such as soluble hormones and growth factors, and properties such as stiffness (Fazelian-Dehkordi *et al.*, 2022; Wu *et al.*, 2022b). To address these problems, it is suggested that dECM undergo enzyme-linked immunosorbent assay (ELISA), stress relaxation testing and atomic force microscopy. Ovaries are important endocrine glands that secrete estrogen, progesterone, and anti-Müllerian hormone (Leong, 2018). Nevertheless, the amounts of these components within dECM scaffolds remain obscure although they are expected to facilitate the growth of seeding cells. Recently, ECM mechanical cues were demonstrated to underlie the development of polycystic ovary syndrome and *in vitro* activation (Lunde *et al.*, 2001; Wood *et al.*, 2015; Kawamura *et al.*, 2016). ECM accumulation along with aging leads to a fibrotic ovary and worsens follicle development (Shah *et al.*, 2018), which underlines the fact that dECM rigidity should be tested and modified for artificial ovary construction (Chiti *et al.*, 2018).

The dECM-derived artificial ovaries demonstrate great potential for restoring ovarian function. In most of the included studies, preantral follicles formed the antral cavity, underwent maturation, and produced estradiol after reseeding on the dECM scaffolds. Many groups achieved healthy follicle development both *in vitro* and *in vivo*. Nevertheless, further progress concerning any pregnancy or live birth of experimental animals with the dECM scaffolds has never been reported, which would be a milestone in the field of decellularized materials. Additionally, more in-depth studies concerning safety are needed.

### Future perspectives

In addition to direct application as solid platforms, dECM-based materials can also be produced via 3D printing and microfluidic chips. Such bioengineering approaches have recently been applied in the vagina and for ovary regeneration (Hou *et al.*, 2021; Zheng *et al.*, 2022). After a series of lyophilization, pulverization, digestion, and solubilization, the dECM powder is formed into a hydrogel, serving as a printable bioink (Kim *et al.*, 2019). Meanwhile, the rheological, flow and gelatin behaviors of the bioinks are characterized to aid parameter optimization during printing (Das *et al.*, 2019). The bioink extrusion speed, nozzle routing, strut distance, and layer height should be coordinated. Finally, the dECM hydrogel is shaped via 3D printing and a biomimetic ovary mimicking the actual cell arrangement is precisely fabricated (Laronda *et al.*, 2017). Mixing dECM hydrogel with collagens is expected to create a more rigid environment similar to that of ovary fibrosis and the cortex of polycystic ovary syndrome (Filatov *et al.*, 2015; Ouni *et al.*, 2020; Kim *et al.*, 2021a). The combination of dECM and hyaluronic acid is suitable for the survival of the cumulus cell-oocyte complex, which is essential for ovulation and fertilization (Briggs *et al.*, 2015; Serati *et al.*, 2015). When integrating with microfluidic chips, the decellularization and recellularization manipulation can be reproduced directly in the devices or used as medium to fill the chips (Hong *et al.*, 2017; Palikuqi *et al.*, 2020; Bhatt *et al.*, 2022). The latter provides a dynamic stimulus to the cumulus cell–oocyte complex or denuded oocytes, which improves the outcomes of oocyte maturation and IVF (Nagashima *et al.*, 2018; Podwin *et al.*, 2020; Healy *et al.*, 2021; Sadeghzadeh Oskouei *et al.*, 2021). Altogether, the integration of these advanced culture systems will facilitate the development of ovarian regeneration and drug-testing models.

Even though decellularization eliminates most immunogenic substances, adverse effects that include inflammatory reactions, fibrosis and calcification have been recorded (Padalino *et al.*, 2016; Woo *et al.*, 2016; Hofmann *et al.*, 2017). Most studies did not focus adequately on evaluating the residual antigenicity. α galactosidase (αGal) is the major xenogeneic antigen that causes rejection-related responses, but no included studies examined the αGal level (Li *et al.*, 2021). It is also suggested that dECM is elevated for the release of interleukins, chemokines and other cytokines; therefore, blockade intervention is recommended in xenotransplantation (Islam *et al.*, 2021). Antigenicity is also associated with the decellularization protocols. Prolonged operation times and increased detergent concentrations are used to reinforce decellularization efficiency. However, these adjustments may further expose the hidden antigenic motifs in ECM components, such as laminin, aggrecan, versican, collagen types I and IV, and hyaluronan (Chakraborty *et al.*, 2020). The 7S domain constitutes the amino-terminal end of type IV collagen; when exposed after decellularization, it will cause a neutrophil chemotactic response (Senior *et al.*, 1989). Hyaluronan is an abundant ECM component enriched in follicular fluid and ovarian stroma. However, it also has diverse roles in chronic inflammation and immune cell activation and can even cause autoimmune diseases (Johnson *et al.*, 2018; Nagy *et al.*, 2019). The matrikines refer to a group of ECM fragments and are inactive in most cases. However, structural and conformational alterations in ECM proteins may result in matrikine release by proteolysis, which contributes to fibrosis, cancer, and aging (Abdul Roda *et al.*, 2015; Jariwala *et al.*, 2022). In some circumstances, these minor alterations occur in ECM ultrastructure and cannot be observed via histological staining or electrical microscopy. In such cases, thermal analysis characterization using differential scanning calorimetry can be used (Samouillan *et al.*, 1999; Wu *et al.*, 2022b). To summarize, appropriate selection criteria are a prerequisite to identify the antigenic motif or matrikine on xenogeneic decellularized tissue to avoid the possibility of interspecies reaction upon clinical application.

## Conclusion

To our knowledge, this is the first systematic review to provide a broad overview on the current state-of-the-art of dECM-based-artificial ovaries. Artificial ovaries provide promising opportunities to restore ovarian function, yet animal studies and preclinical applications of dECM-derived artificial ovaries have only just begun. It is important to comprehensively assess the decellularized scaffolds and demonstrate their effectiveness. Standardizing decellularization protocols and the implementation of quality controls and cytotoxicity measurements will enable the construction of generic and comparable dECM-based artificial ovaries in the future.

## Supplementary Material

hoad014_Supplementary_Data

## Data Availability

All data used for the study have been included in the article and Supplementary Material.
