# Supplementary material for: Extracellular matrix-derived scaffolds in constructing artificial ovaries for ovarian failure: a systematic methodological review
Source: Hum Reprod Open. 2023 Apr 20;2023(2):hoad014. doi: 10.1093/hropen/hoad014 (PMC10174707; doi:10.1093/hropen/hoad014)
Supplement: hoad014_Supplementary_Data [file hoad014_supplementary_data.docx]

| **Supplementary Table S1. Specific search terms of databases (October 20, 2022)** | | |
| --- | --- | --- |
| **Database** | **Search term** | **Number** |
| **PubMed** | ((((ovary) OR (ovarian tissue)) OR (follicle)) AND ((((decellularization) OR (acellular))) OR (recellularization))) AND (English[Language]) | 144 |
| **Embase** | (decellularized or acellular or decellularization).ab,dm,dq,dv,fx,hw,kf,mf,ti,tn. AND (ovary or ovarian tissue or ovarian follicle).ab,dm,dq,dv,fx,hw,kf,mf,ti,tn. | 377 |
| **Web of Science** | (TS=(decellularization) OR TS=(acellular) OR TS=(recellularization)) AND ((TS=(ovary) OR TS=(ovarian tissue) OR TS=(ovarian follicle))) | 209 |
| **Cochrane CENTRAL** | ((decellularization):ti,ab,kw OR (acellular):ti,ab,kw OR (recellularization):ti,ab,kw (Word variations have been searched)) AND ((ovary):ti,ab,kw OR (ovarian tissue):ti,ab,kw OR (ovarian follicle):ti,ab,kw (Word variations have been searched)) | 24 |

| **Supplementary Table S2. Included and excluded studies in the systematic review.** | | | | |
| --- | --- | --- | --- | --- |
| **Order** | **Study information** | **Type** | **Status** |  |
| 1 | Alaee, S., et al. (2021). "The decellularized ovary as a potential scaffold for maturation of preantral ovarian follicles of prepubertal mice." Syst Biol Reprod Med 67(6): 413-427. | Research | Included |  |
| 2 | Alshaikh, A. B., et al. (2019). "Decellularization of the mouse ovary: comparison of different scaffold generation protocols for future ovarian bioengineering." J Ovarian Res 12. | Research | Excluded: no ovarian cell |  |
| 3 | Alshaikh, A. B., et al. (2020). "Decellularization and recellularization of the ovary for bioengineering applications; studies in the mouse." Reproductive Biology and Endocrinology 18(1). | Research | Excluded: no ovarian cell |  |
| 4 | Brown, B., et al. Hydrogel useful for maturing oocyte, preserving fertility, and remodeling or repairing damaged tissue comprises enzymatically digested decellularized ovarian tissue, Univ Pittsburgh Commonwealth System High. | Patent | Excluded: no control group |  |
| 5 | Buckenmeyer, M. J., et al. (2017). "Ovarian ECM-derived hydrogels provide a bioactive scaffold for follicle maturation." Tissue Engineering Part A 23: S15-S15. | Meeting | Excluded: repeated in No.4 |  |
| 6 | Campo, H., et al. (2021). "Decellularization methods of ovary in tissue engineering." Adv Exp Med Biol 1345: 129-139. | Review | Excluded: review article |  |
| 7 | Chiti, M. C., et al. (2022). "Ovarian extracellular matrix-based hydrogel for human ovarian follicle survival in vivo: A pilot work." J Biomed Mater Res B Appl Biomater 110(5): 1012-1022. | Research | Included |  |
| 8 | Chiti, M. C., et al. (2020). "Hydrogel derived from decellularized bovine ovarian extracellular matrix supports human follicle survival in vitro." Human Reproduction 35: I325-I325. | Meeting | Excluded: repeated in No.7 |  |
| 9 | Damous, L. L., et al. (2015). "Scaffold-based delivery of adipose tissue-derived stem cells in rat frozen-thawed ovarian autografts: preliminary studies in a rat model." J Assist Reprod Genet 32(8): 1285-1294. | Research | Excluded: not decellularized scaffold |  |
| 10 | Eivazkhani, F., et al. (2019). "Evaluating two ovarian decellularization methods in three species." Mater Sci Eng C Mater Biol Appl 102: 670-682. | Research | Excluded: no control group |  |
| 11 | Fazelian-Dehkordi, K., et al. (2022). "Three-dimensional in vitro maturation of rabbit oocytes enriched with sheep decellularized greater omentum." Vet Med Sci 8(5): 2092-2103. | Research | Included |  |
| 12 | Frances-Herrero, E., et al. (2022). "Enhancing development of murine follicles in vitro using extracellular matrix from decellularized bovine ovarian cortex." Reproductive Sciences 29(SUPPL 1): 76-76. | Meeting | Excluded: protocol not described |  |
| 13 | Gandolfi, F., et al. (2020). "Bioengineering the ovary to preserve and reestablish female fertility." Anim Reprod 16(1): 45-51. | Review | Excluded: review article |  |
| 14 | Haghshenas, Mohammad et al. "Mouse ovarian follicle growth in an amniotic membrane-based hydrogel." J Biomater Appl | Research | Included |  |
| 15 | Hassanpour, A., et al. (2018). "Decellularized human ovarian scaffold based on a sodium lauryl ester sulfate (SLES)-treated protocol, as a natural three-dimensional scaffold for construction of bioengineered ovaries." Stem Cell Res Ther 9. | Research | Included |  |
| 16 | Henning, N. F., et al. (2019). "Proteomic analyses of decellularized porcine ovaries identified new matrisome proteins and spatial differences across and within ovarian compartments." Sci Rep 9. | Research | Excluded: no ovarian cell |  |
| 17 | Jain, A. and R. Bansal (2015). "Applications of regenerative medicine in organ transplantation." J Pharm Bioallied Sci 7(3): 188-194. | Review | Excluded: review article |  |
| 18 | Jakus, A. E., et al. (2017). "Tissue papers" from organ-specific decellularized extracellular matrices." Adv Funct Mater 27(34). | Research | Excluded: no control group |  |
| 19 | Jakus, A. E. and R. N. Shah Porous scaffold for use as substrates for attachment, growth and proliferation of variety of cells, has decellularized extracellular matrix particles and elastomer, where scaffold is planar including specific thickness, Univ Northwestern. | Patent | Excluded: repeated in No.17 |  |
| 20 | Khazaei, M., et al. (2021). "Functional survey of decellularized tissues transplantation for infertile females." Cell Tissue Bank. | Review | Excluded: review article |  |
| 21 | Kim, S. W., et al. (2021). "Recent advancements in engineered biomaterials for the regeneration of female reproductive organs." Reprod Sci 28(6): 1612-1625. | Review | Excluded: review article |  |
| 22 | Laronda, M. M., et al. (2015). "Initiation of puberty in mice following decellularized ovary transplant." Biomaterials 50: 20-29. | Research | Included |  |
| 23 | Li, Q., et al. (2022). "Drug-free in vitro activation combined with 3D-bioprinted adipose-derived stem cells restores ovarian function of rats with premature ovarian insufficiency." Stem Cell Res Ther 13(1): 347. | Research | Excluded: protocol not described |  |
| 24 | Liu, W. Y., et al. (2017). "Xenogeneic decellulratarized scaffold: a novel platform for ovary regeneration." Tissue Eng Part C Methods 23(2): 61-71. | Research | Excluded: no ovarian cell |  |
| 25 | Mayorca-Guiliani, A. E., et al. (2019). "Decellularization and antibody staining of mouse tissues to map native extracellular matrix structures in 3D." Nat Protoc 14(12): 3395-3425. | Research | Excluded: no ovarian cell |  |
| 26 | Mirzaeian, L., et al. (2020). "Optimizing the cell seeding protocol to human decellularized ovarian scaffold: application of dynamic system for bio-engineering." Cell J 22(2): 227-235. | Research | Excluded: no ovarian cell |  |
| 27 | Mitchell, S., et al. Producing decellularized tissue engineered construct (TEC) by decellularizing TEC, and producing decellularized engineered native tissue involves engineering and then decellularizing tissue harvested from human/animal, Univ Duke; Mitchell S; Koh J; Prabhakar V; Niklason L; Niklason L E. | Patent | Excluded: no ovarian cell |  |
| 28 | Motamed, M., et al. (2017). "Tissue engineered human amniotic membrane application in mouse ovarian follicular culture." Ann Biomed Eng 45(7): 1664-1675. | Research | Included |  |
| 29 | Nikniaz, H., et al. (2019). "Primordial follicle viability following culture in to the decellularized ovarian scaffold in vitro." Human Reproduction 34: 357-357. | Meeting | Excluded: repeated in No.27 |  |
| 30 | Nikniaz, H., et al. (2021). "Comparing various protocols of human and bovine ovarian tissue decellularization to prepare extracellular matrix-alginate scaffold for better follicle development in vitro." BMC Biotechnol 21(1): 8. | Research | Included |  |
| 31 | Oneill, J. and G. Vunjak-Novakovic New engineered tissue used for e.g. growing, maintaining, or differentiating organ or region-specific cells in culture, and stimulating tissue repair or regeneration in vivo comprises tissue-derived decellularized extracellular matrix, Univ Columbia New York. | Patent | Excluded: not decellularized scaffold |  |
| 32 | Pennarossa, G., et al. (2021). "Current advances in 3D tissue and organ reconstruction." Int J Mol Sci 22(2). | Review | Excluded: review article |  |
| 33 | Pennarossa, G., et al. (2021). "Ovarian decellularized bioscaffolds provide an optimal microenvironment for cell growth and differentiation in vitro." Cells 10(8). | Research | Included |  |
| 34 | Pennarossa, G., et al. (2022). "Impact of aging on the ovarian extracellular matrix and derived 3D scaffolds." Nanomaterials (Basel) 12(3). | Research | Excluded: no ovarian cell |  |
| 35 | Pennarossa, G., et al. (2020). "Whole-ovary decellularization generates an effective 3D bioscaffold for ovarian bioengineering." J Assist Reprod Genet 37(6): 1329-1339. | Research | Excluded: no control group |  |
| 36 | Pennarossa, G., et al. (2021). "Creation of a bioengineered ovary: isolation of female germline stem cells for the repopulation of a decellularized ovarian bioscaffold." Methods Mol Biol 2273: 139-149. | Research | Excluded: no control group |  |
| 37 | Pennarossa, G., et al. (2023). "Synergistic effect of miR-200 and young extracellular matrix-based bio-scaffolds to reduce signs of aging in senescent fibroblasts." Stem Cell Rev Rep 19(2): 417-429. | Research | Excluded: no ovarian cell |  |
| 38 | Pors, S., et al. (2018). "Towards an artificial ovary: grafting preantral follicles on decellularized human ovarian tissue." Human Reproduction 33: 39-40. | Meeting | Excluded: repeated in No.39 |  |
| 39 | Pors, S. E., et al. (2019). "Initial steps in reconstruction of the human ovary: survival of pre-antral stage follicles in a decellularized human ovarian scaffold." Hum Reprod 34(8): 1523-1535. | Research | Excluded: no control group |  |
| 40 | Sarabadani, M., et al. (2021). "Co-culture with peritoneum mesothelial stem cells supports the in vitro growth of mouse ovarian follicles." J Biomed Mater Res A 109(12): 2685-2694. | Research | Included |  |
| 41 | Shin, E. Y., et al. (2021). "Prevention of chemotherapy-induced premature ovarian insufficiency in mice by scaffold-based local delivery of human embryonic stem cell-derived mesenchymal progenitor cells." Stem Cell Res Ther 12(1): 431. | Research | Excluded: not decellularized scaffold |  |
| 42 | Sistani, M. N., et al. (2021). "Characteristics of a decellularized human ovarian tissue created by combined protocols and its interaction with human endometrial mesenchymal cells." Prog Biomater 10(3): 195-206. | Research | Excluded: no control group |  |
| 43 | Tamadon, A., et al. (2016). "Efficient biomaterials for tissue engineering of female reproductive organs." Tissue Eng Regen Med 13(5): 447-454. | Review | Excluded: review article |  |
| 44 | Taylan, E. and K. Oktay (2018). "Application of decellularized tissue scaffolds in ovarian tissue transplantation." Methods Mol Biol 1577: 177-181. | Research | Excluded: no ovarian cell |  |
| 45 | Wu, T., et al. (2022). "Construction of artificial ovaries with decellularized porcine scaffold and its elicited immune response after xenotransplantation in mice." J Funct Biomater 13(4). | Research | Included |  |
| 46 | Zheng, J., et al. (2022). "Ovary-derived decellularized extracellular matrix-based bioink for fabricating 3D primary ovarian cells-laden structures for mouse ovarian failure correction." Int J Bioprint 8(3): 597. | Research | Included |  |

| **Supplementary Table S3. Risk of bias of each study.** | | | | | | | | | | | |
| --- | --- | --- | --- | --- | --- | --- | --- | --- | --- | --- | --- |
| **Study ID** | **Sequence generation** | **Baseline characteristics** | **Allocation concealment** | **Housing of animals** | **Blinding to interventions** | **Selective outcome assessment** | **Blinding outcome assessor** | **Incomplete data** | **Selective reporting** | **Conflict of interest** |  |
| Alaee *et al.* (2021) | low risk | low risk | unclear | unclear | unclear | unclear | low risk | unclear | low risk | low risk |  |
| Chiti *et al.* (2022) | unclear | low risk | unclear | unclear | unclear | unclear | low risk | low risk | low risk | low risk |  |
| Fazelian *et al.* (2022) | unclear | low risk | unclear | unclear | unclear | unclear | low risk | unclear | low risk | low risk |  |
| Haghshenas *et al.* (2022) | unclear | low risk | unclear | unclear | unclear | unclear | low risk | unclear | low risk | low risk |  |
| Hassanpour *et al.* (2018) | low risk | low risk | unclear | unclear | unclear | low risk | low risk | low risk | low risk | low risk |  |
| Laronda *et al.* (2015) | low risk | unclear | unclear | unclear | unclear | unclear | low risk | unclear | low risk | low risk |  |
| Motamed *et al.* (2017) | unclear | low risk | unclear | unclear | unclear | low risk | low risk | unclear | low risk | unclear |  |
| Nikniaz *et al.* (2021) | unclear | low risk | unclear | unclear | unclear | unclear | low risk | low risk | low risk | low risk |  |
| Pennarossa *et al.* (2021) | unclear | unclear | unclear | unclear | unclear | unclear | low risk | unclear | low risk | low risk |  |
| Sarabadani *et al.* (2021) | low risk | low risk | unclear | unclear | unclear | unclear | low risk | unclear | low risk | low risk |  |
| Wu *et al.* (2022) | low risk | low risk | unclear | unclear | unclear | low risk | low risk | low risk | low risk | low risk |  |
| Zheng *et al.* (2022) | unclear | low risk | unclear | unclear | unclear | low risk | low risk | low risk | low risk | low risk |  |

| **Supplementary Table S4. Information of the eligible studies.** | | | | | | | | | |
| --- | --- | --- | --- | --- | --- | --- | --- | --- | --- |
| **Study ID** | **Country** | **Decellularization procedure** | | | **Evaluation** | | **Pre-clinical design** | | |
|  |  | **Preprocessing** | **Sterility** | **Hydrogel** | **Biocompatibility** | **Other property** | **Seeding species** | **Seeding cell** | **Duration** |
| Alaee *et al.* (2021) | Iran | Not reported | Not reported | Not used | Not reported | SEM, Raman microscope | Mouse | Preantral follicle | 12 d, in vitro |
| Chiti *et al.* (2022) | Belgium | Medulla removal  cut (1 cm^2^) | 0.2% peracetic acid/4% ethanol (2 h) | + | Not reported | SEM, rheological and gelling characteristics | Mouse and human | Preantral follicle | 7 d, in vitro |
| Fazelian *et al.* (2022) | Iran | Cut (2 cm×2 cm)  osmotic shock (24 h)  dehydration (0.5 h)  100% acetone (24 h)  100% ethanol (0.5 h)  70% ethanol (overnight) | Ultraviolet light (0.5 h) | Not used | MTT | SEM | Rabbit | Cumulus-oocyte complex | 24 h, in vitro |
| Haghshenas *et al.* (2022) | Iran | Cut (3–4 cm^2^) | Not reported | + | MTS, live/dead | SEM, rheological characteristics, degradation rate | Mouse | Preantral follicle | 14 d, in vitro |
| Hassanpour *et al.* (2018) | Iran | Medulla removal  cut (2 mm thickness) | Not reported | Not used | MTT, SEM | SEM | Mouse | OSC | 28 d, in vivo |
| Laronda *et al.* (2015) | the Unite States | Medulla removal  cut (0.5 mm thickness) | Ethanol | Not used | Not reported | SEM | Mouse | OSC | 4 w, in vivo |
| Motamed *et al.* (2017) | Iran | Chorion removal  cut (5 cm×5 cm) | Not reported | Not used | MTS | SEM | Mouse | Primary-secondary follicle | 9 d, in vitro |
| Nikniaz *et al.* (2021) | Iran | Medulla removal  cut (0.2 cm)  freeze/thaw | Not reported | Not used | MTT | SEM | Mouse | Preantral follicle | 7 d, in vitro |
| Pennarossa *et al.* (2021) | Italy | Freeze/thaw | 70% ethanol/2% antibiotic solution (0.5 h) | Not used | MTT | Not reported | Porcine and human | OSC and EpiE | 7 d, in vitro |
| Sarabadani *et al.* (2021) | Iran | Freeze/thaw (10 mM Tris base and 5 mM EDTA) | Not reported | Not used | Not reported | SEM | Mouse | Early preantral follicle | 9 d, in vitro |
| Wu *et al.* (2022) | China | Pricking  medulla removal  cut  freeze/thaw | 0.1% peracetic acid/4% ethanol (4 h) | Not used | Ki67, TUNEL | SEM, residual components of organelles | Mouse | OSC and follicles of all stages | 4 w, in vivo |
| Zheng *et al.* (2022) | China | Medulla removal  cut (3 mm thickness) | 0.1% peracetic acid/20% ethanol (2 h) | + | Live/dead | SEM, rheological characteristics | Mouse | OSC | 4 w, in vivo |
| Abbreviations: EpiE, epigenetically erased dermal fibroblast; MTS, 3-(4,5-dimethylthiazol-2-yl)-5-(3-carboxymethoxyphenyl)-2-(4-sulfophenyl)-2H-tetrazolium,inner salt; MTT, 3-(4, 5-dimethylthiazolyl-2)-2,5-diphenyltetrazolium bromide; OSC, ovarian comatic cell; SEM, scanning electron microscope; TUNEL, terminal deoxynucleotidyl transferase-mediated dUTP nick end labeling. | | | | | | | | | |
